# Toxicity and Patient-Reported Outcomes of a Phase 2 Randomized Trial of Prostate and Pelvic Lymph Node Versus Prostate only Radiotherapy in Advanced Localised Prostate Cancer (PIVOTAL)

**DOI:** 10.1016/j.ijrobp.2018.10.003

**Published:** 2019-03-01

**Authors:** David Dearnaley, Clare L. Griffin, Rebecca Lewis, Philip Mayles, Helen Mayles, Olivia F. Naismith, Victoria Harris, Christopher D. Scrase, John Staffurth, Isabel Syndikus, Anjali Zarkar, Daniel R. Ford, Yvonne L. Rimmer, Gail Horan, Vincent Khoo, John Frew, Ramachandran Venkitaraman, Emma Hall

**Affiliations:** ∗ The Institute of Cancer Research, London, United Kingdom; † Clatterbridge Cancer Centre, Wirral, United Kingdom; ‡ The Royal Marsden NHSFT, London, United Kingdom; § UK Radiotherapy Trials Quality Assurance Group, London, United Kingdom; ‖ Ipswich Hospital NHS Trust, Ipswich, United Kingdom; ¶ Division of Cancer and Genetics, Cardiff University and Velindre Cancer Centre, Cardiff, United Kingdom; # Queen Elizabeth Hospital, Birmingham, United Kingdom; ∗∗ Addenbrooke's Hospital, Cambridge, United Kingdom; †† West Suffolk Hospital, Bury St. Edmunds, United Kingdom; ‡‡ Freeman Hospital, Newcastle upon Tyne, United Kingdom

## Abstract

**Purpose:**

To establish the toxicity profile of high-dose pelvic lymph node intensity-modulated radiation therapy (IMRT) and to assess whether it is safely deliverable at multiple centers.

**Methods and Materials:**

In this phase 2 noncomparative multicenter trial, 124 patients with locally advanced, high-risk prostate cancer were randomized between prostate-only IMRT (PO) (74 Gy/37 fractions) and prostate and pelvic lymph node IMRT (P&P; 74 Gy/37 fractions to prostate, 60 Gy/37 fractions to pelvis). The primary endpoint was acute lower gastrointestinal (GI) Radiation Therapy Oncology Group (RTOG) toxicity at week 18, aiming to exclude a grade 2 or greater (G2+) toxicity-free rate of 80% in the P&P group. Key secondary endpoints included patient-reported outcomes and late toxicity.

**Results:**

One hundred twenty-four participants were randomized (62 PO, 62 P&P) from May 2011 to March 2013. Median follow-up was 37.6 months (interquartile range [IQR], 35.4-38.9 months). Participants had a median age of 69 years (IQR, 64-74 years) and median diagnostic prostate-specific androgen level of 21.6 ng/mL (IQR, 11.8-35.1 ng/mL). At week 18, G2+ lower GI toxicity-free rates were 59 of 61 (96.7%; 90% confidence interval [CI], 90.0-99.4) for the PO group and 59 of 62 (95.2%; 90% CI, 88.0-98.7) for the P&P group. Patients in both groups reported similarly low Inflammatory Bowel Disease Questionnaire symptoms and Vaizey incontinence scores. The largest difference occurred at week 6 with 4 of 61 (7%) and 16 of 61 (26%) PO and P&P patients, respectively, experiencing G2+ toxicity. At 2 years, the cumulative proportion of RTOG G2+ GI toxicity was 16.9% (95% CI, 8.9%-30.9%) for the PO group and 24.0% (95% CI, 8.4%-57.9%) for the P&P group; in addition, RTOG G2+ bladder toxicity was 5.1% (95% CI, 1.7%-14.9%) for the PO group and 5.6% (95% CI, 1.8%-16.7%) for the P&P group.

**Conclusions:**

PIVOTAL demonstrated that high-dose pelvic lymph node IMRT can be delivered at multiple centers with a modest side effect profile. Although safety data from the present study are encouraging, the impact of P&P IMRT on disease control remains to be established.

**Summary**

In a multicenter phase 2 randomized study, we tested the safety of high-dose pelvic lymph node (PLN) irradiation in advanced localized prostate cancer using intensity-modulated radiation therapy (IMRT) techniques. The addition of PLN produced a similarly low incidence of long term side-effects as prostate only IMRT using both clinician- and patient-reported outcomes. We have therefore developed a phase 3 trial testing the efficacy of PLN IMRT in prostate cancer.

## Introduction

Prostate cancer is the most common cancer among men in the United Kingdom.^1^ Although the majority of patients with newly diagnosed cancer have localized disease, a significant proportion have locally advanced disease, carrying a high risk of pelvic lymph node (LN) involvement. Current treatment for such patients is long-term androgen suppression and radiation therapy, which confers an improvement in overall survival.^2^, ^3^

Regional nodal irradiation provides a survival advantage to patients with localized high-risk breast cancer; however, it is uncertain whether the same effect is seen in prostate cancer.^4^ Two previous randomized controlled trials^5^, ^6^ failed to demonstrate benefit from pelvic LN radiation therapy, but these evaluations used modest radiation therapy doses to the pelvis and had additional methodological problems.^4^ A recent retrospective review of the US National Cancer Data Base^7^ also found no benefit of pelvic irradiation over prostate only, in terms of overall survival; however, patients with higher-risk features were more likely to have received pelvic irradiation. Although intensity-modulated radiation therapy (IMRT) was used in some cases, the pelvic radiation dose was generally conservative, and median follow-up was 81 months; therefore, there might not have been sufficient time to observe an effect on overall survival. The radiation therapy techniques used in these studies have since been superseded by IMRT, enabling better shaping of dose distributions to target volume, reducing bowel irradiation, and allowing dose escalation to the LN.^5^, ^6^

The PIVOTAL trial was designed to establish the toxicity profile of high-dose pelvic LN IMRT and to assess whether it was safely deliverable at multiple centers. Patient-reported outcomes (PROs) were a key secondary endpoint, with a focus on gastrointestinal (GI) symptoms, to assess the effect of high-dose pelvic LN irradiation from the patient's perspective.

## Methods and Materials

### Trial design

PIVOTAL (CRUK/10/022) is a randomized noncomparative multicenter phase 2 trial of prostate-only (PO) versus prostate and pelvic LN (P&P) IMRT for locally advanced prostate cancer. The aims were to assess acute and late toxicity, patient-reported toxicity, and the ability of multiple radiation therapy centers to deliver P&P IMRT per protocol. The trial was registered (ISRCTN48709247), approved by the West Midlands – Edgbaston Multi-centre Research Ethics Committee (10/H1208/54), sponsored by the Institute of Cancer Research, and conducted in accordance with the principles of good clinical practice. All participants provided written informed consent. The Institute of Cancer Research Clinical Trials and Statistics Unit (ICR-CTSU; London, UK) coordinated the study and carried out central statistical data monitoring and all analyses. The trial management group was overseen by an independent trial steering committee. Safety and efficacy data were reviewed regularly by an independent data monitoring committee.

### Patient eligibility and selection

Eligible patients provided written informed consent and had histologically confirmed, previously untreated, localized adenocarcinoma of the prostate, stage T3b/T4 (or a calculated risk of pelvic LN involvement ≥30%)^8^; received luteinizing hormone–releasing hormone (LHRH) analogs for 6 to 9 months before radiation therapy; had normal blood count (Hb>11 g/dL, WBC>4 × 10^9^ cells/L, platelets > 100 × 10^9^ cells/L); were ≥18 years old; and had World Health Organization performance status score of 0 to 1 and prostate-specific antigen level <4 ng/mL before randomization.

Key exclusion criteria were radiologically positive, suspicious, or pathologically confirmed LN involvement; castrate-resistant prostate cancer; other invasive malignancy in the past 5 years (other than basal cell carcinoma); prior pelvic radiation therapy or major pelvic surgery; life expectancy <5 years; bilateral hip prostheses or fixation; or the presence of a comorbid condition likely to affect delivery of pelvic radiation therapy.

### Randomization

Randomization took place by telephone to the trial coordinating center within 8 weeks before start of radiation therapy. Participants were assigned 1:1 between PO and P&P IMRT using computer-generated random permuted blocks of size 4, stratified by radiation therapy center. Treatment allocation was not blinded.

### Treatment

Both treatment groups received 74 Gy in 37 fractions to the prostate and involved seminal vesicles (the contemporaneous standard of care in the United Kingdom). Those in the P&P group received 60 Gy in 37 fractions (1.62-Gy fractions) to the LN regions.

Target and organ-at-risk volumes were defined according to International Commission on Radiation Units guidelines.^9^, ^10^ Mandatory and optimal dose constraints were derived by literature review and defined for rectum, bowel, bladder, and femoral heads.^11^, ^12^, ^13^, ^14^ A vascular expansion technique was developed to identify the LN target with a “bowel expansion” exclusion margin to reduce the inclusion of bowel within the LN planning target volume^15^ so that this was comparable to a previous phase 1/2 study.^5^, ^6^ Target volumes, dose parameters, and optimal and mandatory dose constraints are shown in Appendix A (available online at https://doi.org/10.1016/j.ijrobp.2018.10.003).

If individual plans failed to meet the optimal dose constraints, target volumes and dose distributions were reviewed locally to produce a clinically acceptable option. If mandatory bowel dose constraints were not met, dose to pelvic LN was dropped to 55 Gy in 37 fractions.

IMRT was conducted in accordance with the center's standard technique. Image-guided radiation therapy was permitted, and the minimum treatment verification required was appropriate online–offline imaging 3 times in week 1 of treatment and subsequently at least weekly using online–offline corrections. Fiducial markers were permitted; however, margins applied to create planning target volumes could not be altered. Sites had to use similar treatment protocols for both groups, and all radiation therapy techniques were approved in advance by the trial management group. A pretrial quality assurance program accredited sites for treatment within PIVOTAL, and the plans and outlines for the first 3 P&P participants per site were reviewed by the chief investigator or delegated accredited reviewer.

Treatment was delivered daily for 7.5 weeks. Centers followed local bladder and bowel preparation practice before treatment.

### Assessments

Clinician assessment of acute toxicity was conducted at weeks 2, 4, 6, 8, 10, and 18 from the start of radiation therapy. Radiation Therapy Oncology Group (RTOG)^16^ scoring criteria were used at each timepoint, and Common Terminology Criteria for Adverse Events (CTCAE) version 4^17^ were used at baseline and before radiation therapy. The Gulliford scoring system^18^ was used before radiation therapy and at week 18. All clinician-assessed scoring criteria were collected at months 6, 12, 18, and 24 from the start of radiation therapy. PROs were completed on paper by participants, using the Inflammatory Bowel Disease Questionnaire (IBDQ),^19^ the Vaizey Incontinence Questionnaire,^20^ and the International Prostate Symptom Score (IPSS) questionnaire^21^ at baseline, before radiation therapy, and at weeks 10 and 18 and months 6, 12, 18, and 24 from the start of radiation therapy. Prostate-specific androgen level was measured at each follow-up after treatment, with digital rectal examination as indicated. Patients were followed up annually after the 2-year point for disease-related endpoints.

### Statistical considerations

The primary endpoint was acute RTOG lower GI toxicity at 18 weeks from start of radiation therapy. Secondary endpoints included the ability to deliver 60 Gy in 37 fractions to the pelvis at participating centers, late toxicity (up to 2 years), PROs, biochemical progression-free survival, time to local progression, time to distant metastases, and overall survival. A Simon single-stage design was used based on data from a single-center dose escalation phase 1/2 study.^5^ The RTOG grade ≥2 (G2+) lower GI toxicity-free rate at 18 weeks, which if true would imply that the P&P group did not warrant further investigation, was set at 80%, with an expected rate of 92%. With a 5% one-sided alpha and 80% power, 50 patients in the P&P group were required; if more than 44 patients were toxicity free at 18 weeks, then the 80% lower limit could be ruled out. A 10% noncompliance rate inflated the target sample size to 55 patients in the P&P group. An equal number of patients in the PO group was sought to obtain prospectively collected toxicity data for standard treatment, resulting in a total sample size of 110 patients.

An interim analysis was conducted after 58 patients had complete 18-week assessments. For early stopping, guidance of >10% patients having a 1-week treatment break for toxicity or >1 center being unable to achieve >50% acceptable dose volume constraints for half of their participants was used.

### Analysis methods

The proportion of patients who were toxicity free (ie, reporting at most RTOG lower GI G≤1) at 18 weeks from the start of radiation therapy is presented by treatment group, with 90% confidence intervals (CIs). Kaplan-Meier methods were used to analyze time to first reported G1+ and G2+ CTCAE and RTOG lower GI and bladder toxicity. Patients who were event-free were censored at the date of last clinical assessment of adverse events or death. Two year cumulative proportions are estimated using the Kaplan-Meier method and incorporate events and time at risk to 27 months to account for assessment visits occurring up to 3 months after their expected visit. The Gulliford rectal scoring system was categorized as none, mild, or moderate-to-severe symptoms, and distributions are presented at each assessment time by treatment group. All toxicity data were included, regardless of the timing of the assessment with no imputation for missing data (109 of 124 [88%] patients had all 6 assessments). Standard scoring methodologies were used for PROs.^22^, ^23^, ^24^ Descriptive statistics summarized scores at each assessment. Change in PRO scores was calculated as the total score at each time point minus the pre–radiation therapy score. A clinically significant change was defined as ±7 points for the IBDQ-bowel domain and ±4 points for both the Vaizey and IPSS total scores.^22^, ^23^, ^24^ The proportion of patients showing clinically significant improvement or deterioration are presented for each assessment. Vaizey and IPSS change scores were reversed so that, like IBDQ, positive change scores indicate an improvement in health-related quality of life. Because this was a noncomparative study, no formal statistical comparisons have been made between treatment groups. Analysis was by assigned treatment group, with patients included if they received at least 1 fraction of radiation therapy. Analyses were conducted using STATA version 13.0 (StataCorp, College Station, TX).

## Results

**Fig. 1:**
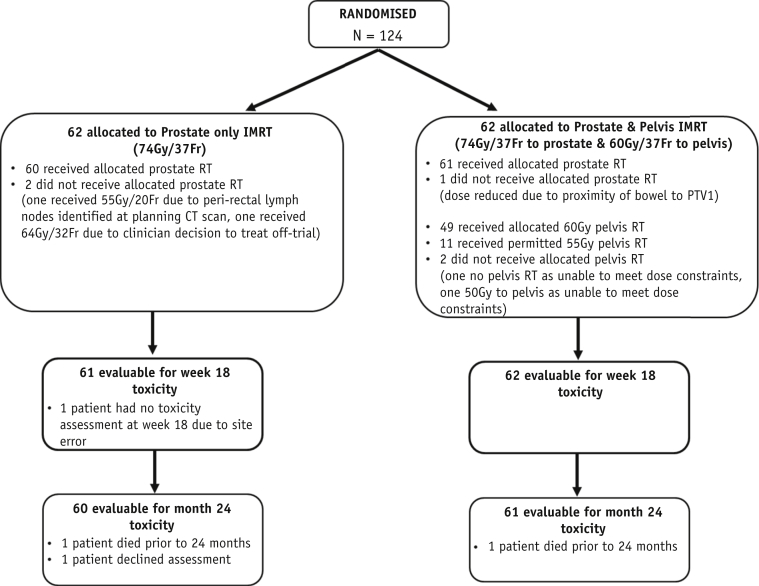
PIVOTAL Consolidated Standards of Reporting Trials flowchart.

One hundred twenty-four participants were randomized (62 PO, 62 P&P) between May 2011 and March 2013 from 9 radiation therapy and 5 referring UK National Health Service Trusts. Median follow-up was 37.6 months (interquartile range, 35.4-38.9 months). Three patients were found to be ineligible after randomization: One had an involved perirectal LN discovered on planning computed tomography scan; 1 had a white blood cell count of 3.3 × 10^9^ cells/L before randomization; and 1 had testosterone >20 ng/dL before randomization. Randomized groups were well balanced for baseline characteristics (Table 1). In the PO group, 57 patients (92%) received LHRH plus short-term antiandrogens, 4 patients (6%) received maximum androgen blockade, and 1 patient received monotherapy bicalutamide. All patients in the P&P group received LHRH with short-term antiandrogens. Median duration of hormone therapy before radiation therapy was 6.8 months in both groups. Adherence to the protocol was good (Fig. 1).

**Table 1:** PIVOTAL patients baseline characteristics by treatment group (n = 124)

Baseline characteristics	Prostate only N = 62 n (%)	Prostate & pelvis N = 62 n (%)	Total N = 124 n (%)
Age, y			
Median (IQR)	68 (65-74)	70 (65-74)	69 (65-74)
Range	54-81	55-81	54-81
Clinical T stage∗			
T1c	1 (2)	1 (2)	2 (2)
T2	31 (50)	30 (48)	61 (49)
T3a	23 (37)	18 (29)	41 (33)
T3b	7 (12)	12 (19)	19 (16)
T4	0	1 (2)	1 (1)
Radiologic T stage			
T1c	0	0	0
T2	15 (25)	17 (30)	32 (26)
T3a	20 (35)	22 (35)	42 (34)
T3b	25 (42)	21 (35)	46 (38)
T4	0	0	0
Not done	2	2	4†
Grade group (Gleason score)			
2 (3 + 4)	13 (21)	8 (13)	21 (17)
3 (4 + 3)	5 (8)	6 (10)	11 (9)
4 (4 + 4, 3 + 5, 5 + 3)	12 (19)	17 (27)	29 (23)
5 (4 + 5, 5 + 4, 5 + 5)	32 (52)	31 (50)	63 (51)
Months from diagnosis to randomization			
Median (IQR)	6.7 (6.1-7.4)	6.6 (5.8-7.6)	6.7 (5.8-7.6)
Range	4.3-10.1	4.1-17.7	4.1-17.7
PSA prediagnostic biopsy			
Mean (SD)	25.2 (19.7)	26.5 (17.3)	25.9 (18.5)
Median (IQR)	21.0 (9.0-34.4)	22.0 (13.3-37.8)	21.6 (11.8-35.1)
Range	0.8-107	4.1-89.5	0.8-107
Number of high-risk‡ features			
1	3 (5)	2 (3)	5 (4)
2	30 (48)	29 (47)	59 (48)
3	29 (47)	31 (50)	60 (48)

*Abbreviations:* IQR = interquartile range; PSA = prostate-specific antigen; SD = standard deviation.

∗ Prehormone magnetic resonance imaging (MRI) scan recommended for staging. Computed tomography (CT) acceptable for lymph node assessment but not assessment of T3b staging. One patient with clinical T3b underwent ultrasound instead of MRI.

† One patient underwent ultrasound imaging, and 1 patient underwent CT rather than MRI. Two patients had unknown reasons for no radiologic staging.

‡ High-risk features: PSA level >20 ng/mL; Grade group 4 or 5 (Gleason score ≥8); radiologic staging ≥T3a.

### Acute toxicity

**Fig. 2:**
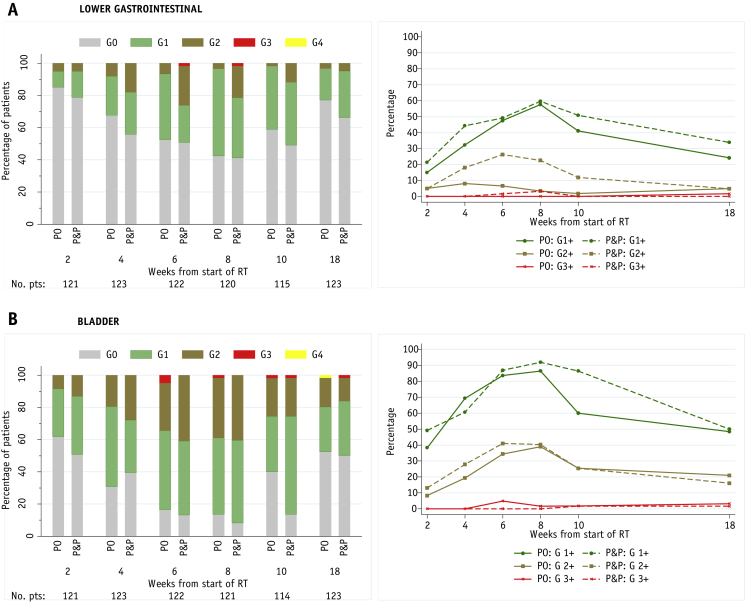
Distribution of acute Radiation Therapy Oncology Group (RTOG) toxicity and prevalence of grade 1+, grade 2+, and grade 3+ toxicity at weeks 2, 4, 6, 8, 10, and 18 from the start of radiation therapy. (A) Lower gastrointestinal symptoms. (B) Bladder symptoms.

No RTOG G4 lower GI acute toxicity events were reported. One patient in the P&P group had RTOG G3 proctitis, diarrhea, and rectal bleeding at 6 and 8 weeks. Patients in the P&P group experienced more G2 toxicity than those in the PO group; the largest difference occurred at week 6 with 4 of 61 patients (7% and 15 of 61 patients (25%) with toxicity assessments in the PO and P&O groups, respectively, experiencing G2 toxicity. This difference declined toward week 18 (Fig. 2A) when 59 of 61 patients in the PO group (96.7%; 90% CI, 90.0%-99.4%) and 59 of 62 patients in the P&P group (95.2%; 90% CI, 88.0%-98.7%) were free of RTOG lower GI G2+ toxicity. The Gulliford score suggested that patients in the P&P group experienced bowel symptoms more commonly than did patients in the PO group at week 18, although no statistical comparisons were made Appendix B.

One patient in the PO group experienced RTOG G4 bladder toxicity (urinary urgency, frequency, and inability to pass urine) at week 18; there were no G4 bladder events reported in the P&P group. G3 bladder toxicity was reported in 5 patients in the PO group and 2 patients in the P&P group during the first 18 weeks from the start of radiation therapy. The prevalence of G2+ bladder toxicity was similar across treatment groups (Fig. 2B), as was G1+ bladder toxicity except for week 10, when 51 of 59 patients (86%) in the P&P group reported any toxicity compared with only 33 of 55 patients (60%) in the PO group. Peak acute toxicity of any grade (lower GI or bladder) occurred at week 8 in both treatment groups.

### Late toxicity

**Fig. 3:**
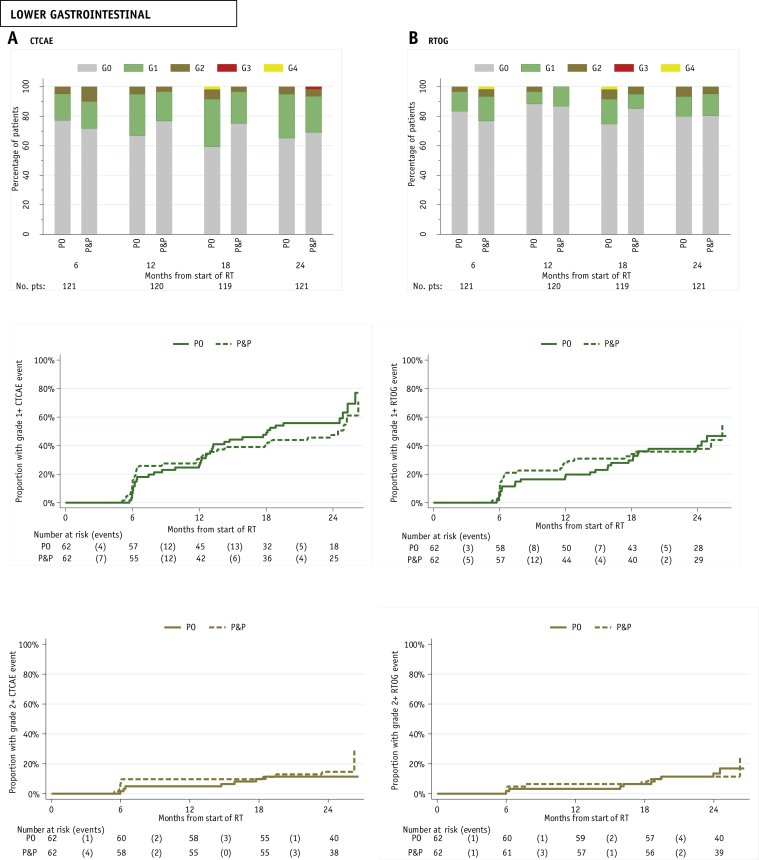

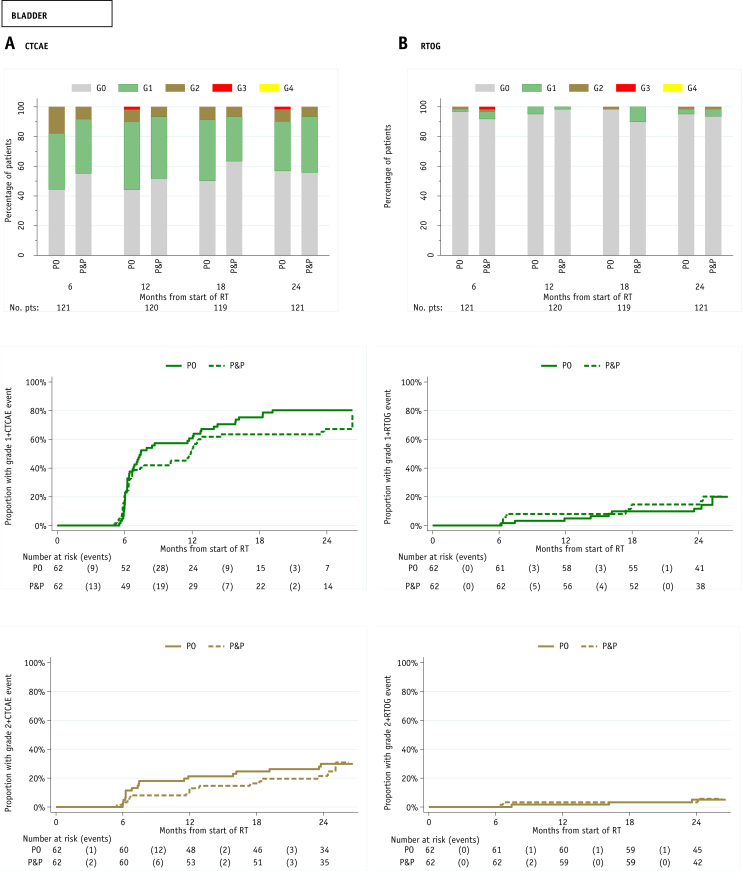
Distribution of late (A) Common Terminology Criteria for Adverse Events (CTCAE) and (B) Radiation Therapy Oncology Group (RTOG) lower gastrointestinal and bladder toxicity and time to first reported G1+ and G2+ toxicity.

Clinician assessments of toxicity up to 24 months indicated very low levels of GI G2+ toxicities according to both the CTCAE and RTOG scoring systems (Fig. 3). There was 1 G4 CTCAE GI event reported at 18 months in the PO group (constipation). Two patients had G4 RTOG bowel toxicity—1 patient in the P&P group at 6 months (bowel obstruction) and 1 patient in the PO group at 18 months (bowel obstruction). One patient in the PO group had CTCAE G3 GI toxicity (proctitis) at 24 months, but no late G3 RTOG bowel toxicity was reported. The cumulative proportion of RTOG G2+ GI toxicity at 2 years was 16.9% (95% CI; 8.9%-30.9%) and 24.0 (95% CI, 8.4%-57.9%) in the PO and P&P groups, respectively. The Gulliford scoring system indicated that at 24 months, the majority of patients experienced no problem with their overall bowel habits (94 of 119), with 8% (10 of 119) reporting a moderate-to-severe problem (Appendix B; available online at https://doi.org/10.1016/j.ijrobp.2018.10.003).

RTOG bladder toxicity was infrequent to 24 months. No G4 bladder toxicity was reported, and 1 patient in the P&P group had a G3 event (cystitis) at 6 months. There was no CTCAE G4 bladder toxicity. Two patients in the PO group had CTCAE G3 bladder toxicity—1 patient (urine incontinence) at 12 months and 1 patient (urine retention) at 24 months. The cumulative proportion of RTOG G2+ bladder toxicity at 2 years was 5.1% (95% CI, 1.7%-14.9%) and 5.6% (95% CI, 1.8%-16.7%) in the PO and P&P groups, respectively.

### Patient-reported outcomes

**Fig. 4:**
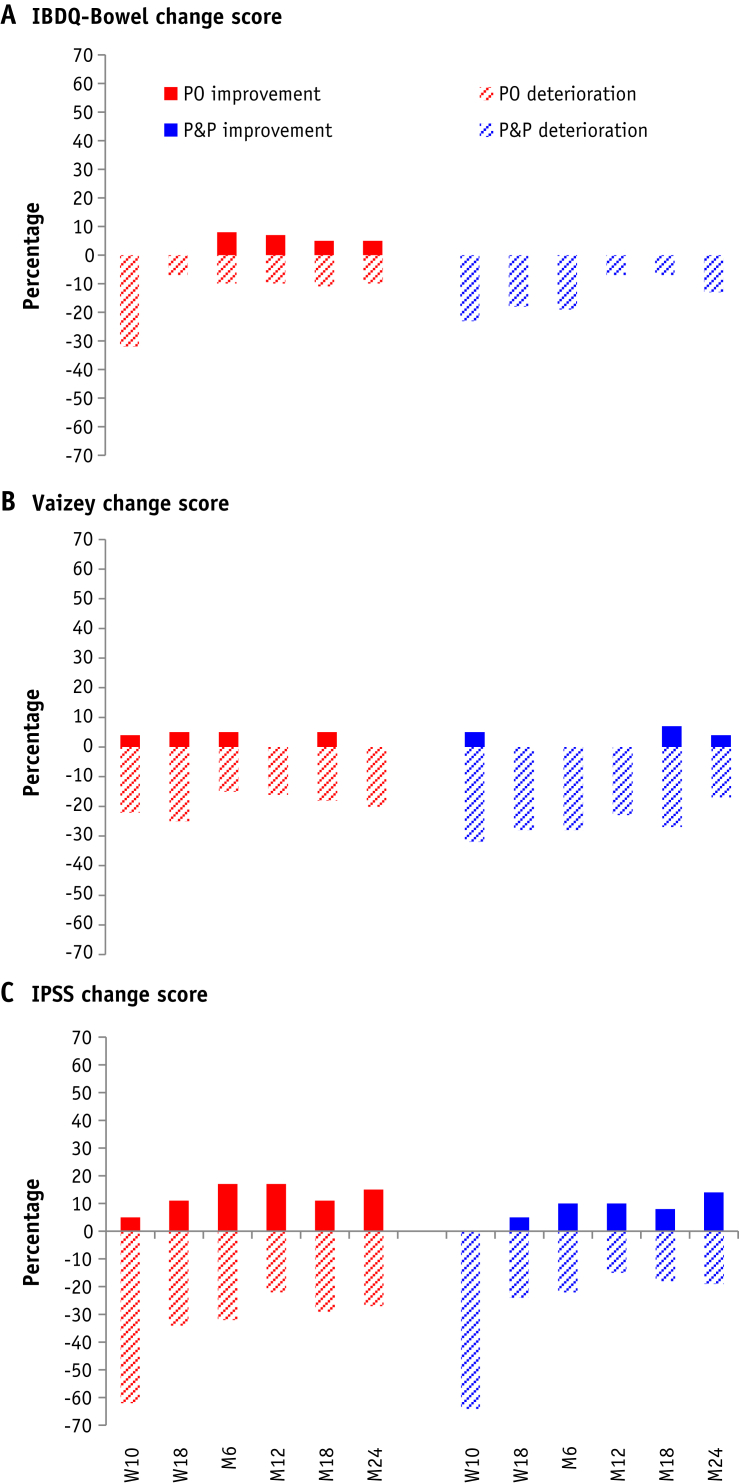
The percentage of patients with clinically significant changes in patient-reported outcomes from pre–radiation therapy to each assessment time for the (A) Inflammatory Bowel Disease Questionnaire (IBDQ) score, (B) Vaizey score, and (C) International Prostate Symptom Score (IPSS) score. An improvement from pre–radiation therapy is indicated as a positive percentage score and a deterioration from pre–radiation therapy is indicated as a negative percentage score.

At week 18, patients reported similar bowel symptoms according to the IBDQ bowel score (Table 2). IBDQ bowel scores remained similar over time for both treatment groups (Table 2). Week 10 had the highest proportion of patients with a clinically significant deterioration in IBDQ bowel score compared with the pre–radiation therapy score in both treatment groups (Fig. 4; Appendixes C and D; available online at https://doi.org/10.1016/j.ijrobp.2018.10.003). Fewer patients in the P&P group had a clinically significant improvement in IBDQ bowel scores up to 24 months compared with patients in the PO group (no statistical comparisons were made). The Vaizey incontinence score indicated similar patient-reported experiences at week 18, with median scores of 3 (interquartile range 0-6) in the PO group and 2 (interquartile range 0-5) in the P&P group. Vaizey incontinence scores remained similar over time for both treatment groups. Patient-reported urinary symptoms were similar at week 18, with 19 of 46 (41%) and 21 of 51 (41%) moderately symptomatic and 6 of 46 (13%) and 5 of 51 (10%) severely symptomatic in the PO and P&P groups, respectively. Week 10 had the greatest proportion of patients with a clinically significant deterioration in IPSS score for both treatment groups. The distribution of change scores appeared similar between randomized groups and did not change greatly over time (Appendix D; available online at https://doi.org/10.1016/j.ijrobp.2018.10.003).

**Table 2:** Summary scores for patient reported IBDQ bowel domain total score, Vaizey total score, IPSS total score, IPSS voiding and storage scores at each time point by treatment group

	Pre-RT		Week 10		Week 18		Month 6		Month 12		Month 18		Month 24	
	PO n (%)	P&P n (%)	PO n (%)	P&P n (%)	PO n (%)	P&P n (%)	PO n (%)	P&P n (%)	PO n (%)	P&P n (%)	PO n (%)	P&P n (%)	PO n (%)	P&P n (%)
IBDQ bowel total score∗														
N	55	55	49	46	47	49	46	51	49	45	44	43	47	48
Median (IQR)	69 (67-70)	69 (67-70)	65 (61-69)	65 (61-68)	68 (65-70)	66 (62-69)	68 (66-69)	68 (62-69)	68 (65-69)	68 (64-70)	67 (64-69)	68 (65-70)	67 (65-69)	68 (67-70)
Range	28-70	60-70	20-70	34-70	48-70	45-70	52-70	49-70	37-70	51-70	42-70	44-70	28-70	44-70
Vaizey Total score†														
N	54	54	50	49	47	52	49	51	49	49	47	43	45	49
Median (IQR)	1 (0-4)	0 (0-3)	2 (0-6)	3 (0-7)	3 (0-6)	2 (0-5)	2 (0-5)	2 (0-5)	2 (1-4)	3 (1-6)	4 (0-6)	1 (0-4)	2 (1-5)	2 (0-5)
Range	0-9	0-8	0-22	0-17	0-17	0-22	0-16	0-17	0-14	0-12	0-18	0-16	0-14	0-16
IPSS														
Mild‡	29 (54)	33 (64)	9 (21)	15 (31)	21 (46)	25 (49)	22 (48)	28 (60)	25 (52)	28 (61)	19 (44)	28 (64)	24 (52)	26 (53)
Moderate	20 (37)	18 (35)	22 (50)	21 (44)	19 (41)	21 (41)	17 (37)	14 (30)	17 (35)	16 (35)	19 (44)	14 (32)	17 (37)	19 (39)
Severe	5 (9)	1 (2)	13 (30)	12 (25)	6 (13)	5 (10)	7 (15)	5 (11)	6 (13)	2 (4)	5 (12)	2 (5)	5 (11)	4 (8)
Total score§														
N	54	52	44	48	46	51	47	48	48	48	43	44	46	49
Median (IQR)	7 (4-11)	6 (3-11)	11 (8-21)	12 (6-19)	9 (5-14)	8 (4-15)	8 (4-12)	6 (3-12)	7 (4-12)	6 (3-13)	8 (4-14)	5 (3-12)	7 (5-12)	6 (3-11)
Range	1-26	0-20	1-33	0-29	1-30	0-22	1-31	0-29	1-24	0-31	2-33	1-22	1-28	1-23
Voiding‖ score														
Median (IQR)	3 (1-5)	1 (0-4)	5 (3-10)	4 (2-9)	3 (1-7)	2 (0-5)	3 (0-5)	2 (0-5)	2 (1-5.5)	2 (0-4)	3 (1-6)	2 (0-4)	3 (1-5)	1 (0-4)
Range	0-18	0-10	0-20	0-17	0-18	0-14	0-18	0-18	0-14	0-17	0-18	0-10	0-16	0-14
Storage¶ score														
Median (IQR)	4.5 (3-7)	4 (2-7)	7 (5-10)	7 (4-11)	5 (4-8)	5 (2-8)	6 (3-8)	4 (3-8)	5 (3-7)	4 (2-8)	5 (3-7)	3 (2-6.5)	5 (3-7)	4 (2-8)
Range	0-12	0-15	1-14	0-15	1-12	0-14	1-14	0-14	1-12	0-15	1-15	1-12	1-12	1-15

*Abbreviations:* IBDQ = Inflammatory Bowel Disease Questionnaire; IPSS = International Prostate Symptom Score; IQR = interquartile range; P&P = prostate and pelvic lymph node group; PO = prostate-only group.

∗ IBDQ bowel domain total score ranges from 0 (most severe symptoms) to 70 (asymptomatic).

† Vaizey total scores ranges from 0 (asymptomatic) to 24 (most severe symptoms).

‡ IPSS score categorized as: mild = 0 to 7; moderate = 8 to 19; severe = 20 to 35.

§ IPSS total score ranges from 0 (asymptomatic) to 35 (most severe symptoms).

‖ IPSS voiding score calculated as total score of incomplete emptying, intermittency, weak stream, and straining.

¶ IPSS storage score calculated as total score of frequency, urgency, and nocturia.

### Disease-related outcomes

Thirteen patients had biochemical progression: 6 in the PO group and 7 in the P&P group. Three patients have recommenced hormones, 2 in the PO group and 1 in the P&P group. Six patients had local recurrence (2 PO and 4 P&P); 1 patient in the PO group had nodal recurrence, and 7 metastatic recurrences were reported (4 PO and 3 P&P). Two patients in the PO group died of prostate cancer. Five patients died of other causes (1 PO, 4 P&P): respiratory causes (2), lung cancer, glioblastoma, ischemic heart disease, and myocardial infarction.

## Discussion

To our knowledge, PIVOTAL is the first randomized controlled trial to assess toxicity prospectively in patients receiving prostate-only and prostate and high-dose pelvic LN IMRT. The study was derived from a single-center trial that reported the development of P&P IMRT techniques and safe dose escalation to pelvic LNs of doses up to 60 Gy/37 fractions.^5^, ^6^ The proportion of patients who received P&P LN IMRT and were free of acute lower GI G2+ toxicity exceeded the predefined threshold of 80% at week 18, and assessment of late adverse effects to 2 years does not indicate concerns about the safety of high-dose P&P LN IMRT.

The pelvic LN dose was approximately 7 to 10 Gy higher than that used in previous studies.^25^, ^26^ Assuming an α/β ratio of 3 Gy (1.5 Gy, 5.0 Gy), our trial gave an equivalent dose at 2 Gy per fraction of 55.4 Gy (53.5 Gy, 56.7 Gy) to the pelvic nodal regions, compared with 48.4 Gy (47.5 Gy, 49 Gy) and 46 Gy (46 Gy, 46 Gy) in the RTOG and Unicancer Genitourinary Group^25^ studies, respectively.

Overall toxicity for the PO group was similar to that of the 74-Gy cohort reported in the Conventional or Hypofractionated High dose intensity modulated radiotherapy for Prostate cancer trial,^11^, ^27^ and toxicity in the P&P group was comparable to results of the single-center pilot study.^5^, ^6^ Acute G1/G2 bladder toxicity was similar in P&P and PO groups, with peak reactions at 6 to 8 weeks and declining by week 18, when there were no differences between the randomized groups. Although acute G2 lower GI toxicity was higher in the P&P group than in the PO group from weeks 4 to 10, there was no difference between the randomized groups by week 18. Rates of G2+ acute and late toxicity were similar to, and in some cases lower than, those observed in other trials investigating prostate radiation therapy that did not include pelvic irradiation.^28^ The favorable side effect profile might relate to the pelvic LN contouring method and bowel constraints mandated in the trial.

The cumulative proportion and prevalence of late GI toxicity was similar in the 2 randomized groups. There was no suggestion of an increasing toxicity profile over time in either group. The CTCAE GI adverse effects followed a similar pattern. Bladder late adverse effects reported on the RTOG scale were low, with no difference between the randomized groups. Although the CTCAE assessment showed no differences between the randomized groups, reported scores were higher than those on the RTOG scale. This has been noted previously by other investigators^29^ and may relate to the inclusion of pretreatment symptoms. The RTOG assessment seems more in keeping with the stability of the pretreatment and posttreatment IPSS scores.

The IBDQ and Vaizey questionnaires yielded similar results for both treatment groups up to 2 years. The 10-week time point showed the worst symptoms for the IBDQ questionnaire, which is similar to the clinician-reported data showing the worst symptoms at 8 weeks. Our results demonstrate that the majority of patients have little change to their bowel function from week 18 to month 24 in comparison with function before radiation therapy. The findings of the IBDQ suggest that the P&P group experienced a greater severity of bowel morbidity in a few patients, which is similar to the clinician assessment using the RTOG toxicity score. The advantage of using PROs in addition to clinician assessments is that PROs relate to the impact of the symptom on a particular patient's function and quality of life; therefore, although a toxicity might be graded highly in a clinician-based toxicity score, it might not particularly bother a patient, and vice versa. It is reassuring that the physician and PRO assessments of toxicity are low at 2 years, with the combined assessments giving greater credence to the safety of the P&P treatment.

The safety data from the present study are encouraging; however, any effect of high-dose pelvic LN irradiation on disease control has yet to be established. The recently opened PIVOTALboost trial (ISRCTN80146950, CRUK/16/018) investigates the value of pelvic IMRT as well as the effects of a focal intraprostatic boosts to dominant lesions This will complement other ongoing phase 3 studies, RTOG 09-24 (NCT01368588) and PEACE 2 (NCT01952223), and should finally determine the role of pelvic LN radiation therapy in prostate cancer. An increase in efficacy will need to be demonstrated to offset the small but expected adverse effects of pelvic IMRT.

## Supplementary Data

Appendices A-E
