# Supplementary material for: Toxicity and Patient-Reported Outcomes of a Phase 2 Randomized Trial of Prostate and Pelvic Lymph Node Versus Prostate only Radiotherapy in Advanced Localised Prostate Cancer (PIVOTAL)
Source: Int J Radiat Oncol Biol Phys. 2019 Mar 1;103(3):605–17. doi: 10.1016/j.ijrobp.2018.10.003 (PMC6361768; doi:10.1016/j.ijrobp.2018.10.003)
Supplement: Appendices A-E [file mmc1.docx]

Appendix A

| Tumour and Clinical Target Volumes | Planning Target Volumes | Target Volumes |
| --- | --- | --- |
| Prostate and seminal vesicles: TPSV | PTVpsv_6000  Outline: prostate & seminal vesicles  Margins: 10mm in all directions | Prescribed dose to PTV(psv)-PTV(p2):≥ 60Gy (81%)  Minimum dose:≥ 57Gy (77%) |
| Prostate (+ any involved seminal vesicle): TP | PTVp_7100  Outline: prostate and any involved seminal vesicle  Margins: 10mm except 5mm towards rectum | Prescribed dose to PTV(p2)-PTV(p3):≥71Gy (96%)  Minimum dose:≥ 67.3Gy (91%) |
|  | PTVp_7400  Outline: prostate and any involved seminal vesicle  Margin: 5mm except 0mm towards rectum | Prescribed dose to PTV(p3):  74Gy (100% ± 1%)  Minimum dose:≥ 70.3Gy (95%)  Maximum dose:77.7Gy (105%) |
| Pelvic lymph nodes: CTVN | PTVn_6000  Outline: pelvic lymph nodes  Margin: 5mm in all directions | Prescribed dose to PTV(ln):  ≥60Gy (81%)  Minimum dose:≥57Gy (77%) |

Table A1: Target volumes, planning target margins and planning objectives

| Organ at risk | Dose-volume constraints | | |
| --- | --- | --- | --- |
|  |  | Optimal | **Mandatory** |
| Bowel | V45 | 78cc | **158cc** |
|  | V50 | 17cc | **110cc** |
|  | V55 | 14cc | **28cc** |
|  | V60 | 0.5cc | **6cc** |
|  | V65 | 0cc | **0cc** |
| Rectum | V30 | 80% | **-** |
|  | V40 | 65% | **-** |
|  | V50 | 50% | **60%** |
|  | V60 | 35% | **50%** |
|  | V65 | 30% | **30%** |
|  | V70 | 15% | **15%** |
|  | V75 | 3% | **5%** |
| Bladder | V50 | 50% | **-** |
|  | V60 | 25% | **-** |
|  | V65 | - | **50%** |
|  | V70 | 5% | **35%** |
| Femoral Heads | V50 | 5% | **25%** |

Table A2: Organ at risk dose constraints

Appendix B: Gulliford scoring criteria categorised as none, mild or moderate/severe symptoms at week 18 and months 6, 12, 18 and 24

|  | **Pre-RT** | | **Week 18** | | **Month 6** | | **Month 12** | | **Month 18** | | **Month 24** | |
| --- | --- | --- | --- | --- | --- | --- | --- | --- | --- | --- | --- | --- |
|  | **PO**  **N=60** | **P&P**  **N=59** | **PO**  **N=61** | **P&P**  **N=62** | **PO**  **N=61** | **P&P**  **N=61** | **PO**  **N=60** | **P&P**  **N=60** | **PO**  **N=59** | **P&P**  **N=60** | **PO**  **N=60** | **P&P**  **N=61** |
| **Rectal bleeding**  **None**  **Mild**  **Moderate / Severe** | 53 (88)  5 (8)  2 (3) | 57 (97)  2 (3)  0 | 60 (98)  1 (2)  0 | 55 (89)  6 (10)  1 (2) | 58 (95)  2 (3)  1 (2) | 60 (98)  1 (2)  0 | 56 (93)  4 (7)  0 | 54 (90)  6 (10)  0 | 52 (88)  6 (10)  1 (2) | 52 (87)  7 (12)  1 (2) | 49 (82)  10 (17)  1 (2) | 53 (87)  7 (12)  1 (2) |
| **Proctitis^1^**  **None**  **Mild**  **Moderate/Severe** | -  -  - | -  -  - | 57 (93)  4 (7)  0 | 57 (92)  5 (8)  0 | 56 (93)  4 (7)  0 | 55 (90)  6 (10)  0 | 55 (92)  5 (8)  0 | 53 (88)  7 (12)  0 | 51 (86)  8 (14)  0 | 54 (90)  6 (10)  0 | 52 (87)  7 (12)  1 (2) | 54 (89)  6 (10)  1 (2) |
| **Stool frequency**  **None**  **Mild**  **Moderate / Severe** | 50 (83)  10 (17)  0 | 50 (86)  8 (14)  0 | 48 (79)  13 (21)  0 | 40 (66)  19 (31)  2 (3) | 47 (78)  13 (22)  0 | 38 (63)  21 (35)  1 (2) | 45 (76)  13 (22)  1 (2) | 44 (73)  15 (25)  1 (2) | 44 (76)  13 (22)  1 (2) | 45 (78)  12 (21)  1 (2) | 45 (75)  14 (23)  1 (2) | 45 (76)  14 (24)  0 |
| **Difficulty with sphincter control**  **None**  **Mild**  **Moderate / Severe** | 57 (97)  2 (3)  0 | 57 (97)  2 (3)  0 | 58 (95)  3 (5)  0 | 56 (90)  5 (8)  1 (2) | 57 (97)  2 (3)  0 | 52 (87)  8 (13)  0 | 58 (97)  1 (2)  1 (2) | 56 (95)  3 (5)  0 | 53 (90)  5 (9)  1 (2) | 52 (88)  7 (12)  0 | 58 (97)  2 (3)  0 | 52 (85)  8 (13)  1 (2) |
| **Loose/liquid stool frequency**  **None**  **Mild**  **Moderate / Severe** | 60 (100)  0  0 | 54 (92)  2 (3)  3 (5) | 57 (93)  2 (3)  2 (3) | 50 (83)  6 (10)  4 (7) | 54 (92)  3 (5)  2 (3) | 52 (88)  6 (10)  1 (2) | 55 (92)  2 (3)  3 (5) | 57 (97)  1 (2)  1 (2) | 53 (91)  1 (2)  4 (7) | 52 (88)  2 (3)  5 (9) | 54 (92)  4 (7)  1 (2) | 56 (93)  2 (3)  2 (3) |
| **Rectal urgency frequency**  **None**  **Mild**  **Moderate / Severe** | 56 (93)  3 (5)  1 (2) | 57 (97)  1 (2)  1 (2) | 54 (89)  5 (8)  2 (3) | 48 (79)  7 (11)  6 (10) | 52 (87)  5 (8)  3 (5) | 49 (82)  9 (15)  2 (3) | 51 (85)  4 (7)  5 (8) | 54 (92)  4 (7)  1 (2) | 50 (86)  5 (9)  3 (5) | 47 (81)  9 (16)  2 (3) | 54 (92)  3 (5)  2 (3) | 49 (82)  8 (13)  3 (5) |
| **Overall bowel habit problem**  **None**  **Mild**  **Moderate / Severe** | 52 (88)  7 (12)  0 | 53 (90)  5 (9)  1 (2) | 50 (82)  7 (11)  4 (7) | 45 (75)  13 (22)  2 (3) | 46 (78)  11 (19)  2 (3) | 46 (75)  12 (20)  3 (5) | 46 (77)  10 (17)  4 (7) | 48 (81)  10 (17)  1 (2) | 42 (72)  12 (21)  4 (7) | 43 (74)  10 (17)  5 (9) | 49 (82)  6 (10)  5 (8) | 45 (76)  9 (15)  5 (9) |

^1^ Proctitis not collected prior to radiotherapy

**Appendix C: Clinically significant changes from pre-RT score in IBDQ bowel, Vaizey and IPSS scores at each time point**

|  | **Week 10** | | **Week 18** | | **Month 6** | | **Month 12** | | **Month 18** | | **Month 24** | |
| --- | --- | --- | --- | --- | --- | --- | --- | --- | --- | --- | --- | --- |
|  | **PO** | **P&P** | **PO** | **P&P** | **PO** | **P&P** | **PO** | **P&P** | **PO** | **P&P** | **PO** | **P&P** |
| **IBDQ Bowel score**  Significant improvement  No significant change  Significant deterioration | 0  30 (68)  14 (32) | 0  33 (77)  10 (23) | 0  38 (93)  3 (7) | 0  36 (82)  8 (18) | 3 (8)  32 (82)  4 (10) | 0  39 (81)  9 (19) | 3 (7)  35 (83)  4 (10) | 0  38 (93)  3 (7) | 2 (5)  31 (84)  4 (11) | 0  37 (93)  3 (7) | 2 (5)  36 (86)  4 (10) | 0  40 (87)  6 (13) |
| **Vaizey score**  Significant improvement  No significant change  Significant deterioration | 2 (4)  34 (74)  10 (22) | 2 (5)  28 (64)  14 (32) | 2 (5)  28 (70)  10 (25) | 0  34 (72)  13 (28) | 2 (5)  32 (80)  6 (15) | 0  33 (72)  13 (28) | 0  36 (84)  7 (16) | 0  34 (77)  10 (23) | 2 (5)  30 (77)  7 (18) | 3 (7)  27 (66)  11 (27) | 0  32 (80)  8 (20) | 2 (4)  37 (79)  8 (17) |
| **IPSS score**  Significant improvement  No significant change  Significant deterioration | 2 (5)  13 (33)  25 (62) | 0  15 (36)  27 (64) | 4 (11)  21 (55)  13 (34) | 2 (5)  30 (71)  10 (24) | 7 (17)  21 (51)  13 (32) | 4 (10)  28 (68)  9 (22) | 7 (17)  25 (61)  9 (22) | 4 (10)  31 (76)  6 (15) | 4 (11)  23 (61)  11 (29) | 3 (8)  29 (74)  7 (18) | 6 (15)  23 (58)  11 (27) | 6 (14)  29 (67)  8 (19) |

**APPENDIX D Change in patient reported outcome scores from pre-radiotherapy to week 10, 18, 6, 12, 18 and 24 months from the start of radiotherapy (A – Inflammatory Bowel Disease Questionnaire (IBDQ), B – Vaizey Incontience Questionnaire, C – International Prostate Symptom Score (IPSS))**

**C – IPSS total score**

**B – VAIZEY total score**

**A – IBDQ bowel domain total score**

WEEK 10

WEEK 18

MONTH 6

MONTH 12

MONTH 18

MONTH 24

Red = prostate only, Blue = prostate & pelvis patients. For all plots, a *positive* score indicates an *improvement* from the pre-radiotherapy assessment (NB. Vaizey and IPSS questionnaires are scored in the opposite direction to IBDQ but have been reversed for these plots so that all change scores are in the same direction for all questionnaires). Y-axis range is the largest possible change in score for each of the questionnaires

Appendix E

PIVOTAL Principal Investigators and recruitment by centre (number of patients recruited in bold).

Royal Marsden Hospital, Sutton, Prof David Dearnaley, **22**; Ipswich Hospital, Dr Christopher Scrase, **22**; Queen Elizabeth Hospital, Birmingham, Dr Anjali Zarkar, **12**; Velindre Hospital, Dr John Staffurth, **10**; Royal Marsden Hospital, London, Dr Vincent Khoo, **9**; Heartlands Hospital, Birmingham, Dr Daniel Ford¸**9**; Warrington Hospital, Dr Isabel Syndikus, **9**; Good Hope Hospital, Dr Daniel Ford, **7**; Queen Elizabeth Hospital, King's Lynn, Dr Gayle Horan, **6**; Freeman Hospital, Dr John Frew, **6**; Addenbrooke's Hospital, Dr Yvonne Rimmer, **5**; Clatterbridge Centre for Oncology, Dr Isabel Syndikus, **4**; West Suffolk Hospital, Dr Yvonne Rimmer, **3**.
